# Enzyme engineering: A synthetic biology approach for more effective library generation and automated high-throughput screening

**DOI:** 10.1371/journal.pone.0171741

**Published:** 2017-02-08

**Authors:** Daniela Quaglia, Maximilian C. C. J. C. Ebert, Paul F. Mugford, Joelle N. Pelletier

**Affiliations:** 1 Département de Chimie, Université de Montréal, Montréal, QC, Canada; 2 Center for Green Chemistry and Catalysis (CGCC), Université de Montréal, Montréal, QC, Canada; 3 PROTEO, The Québec Network for Research on Protein Function, Engineering and Applications, Québec, QC, Canada; 4 Département de Biochimie, Université de Montréal, Montréal, QC, Canada; 5 DSM Nutritional Products, 101 Research Drive, Dartmouth, NS, Canada; University of Canterbury, NEW ZEALAND

## Abstract

The *Golden Gate* strategy entails the use of type IIS restriction enzymes, which cut outside of their recognition sequence. It enables unrestricted design of unique DNA fragments that can be readily and seamlessly recombined. Successfully employed in other synthetic biology applications, we demonstrate its advantageous use to engineer a biocatalyst. Hot-spots for mutations were individuated in three distinct regions of *Candida antarctica lipase A* (Cal-A), the biocatalyst chosen as a target to demonstrate the versatility of this recombination method. The three corresponding gene segments were subjected to the most appropriate method of mutagenesis (targeted or random). Their straightforward reassembly allowed combining products of different mutagenesis methods in a single round for rapid production of a series of diverse libraries, thus facilitating directed evolution. Screening to improve discrimination of short-chain versus long-chain fatty acid substrates was aided by development of a general, automated method for visual discrimination of the hydrolysis of varied substrates by whole cells.

## Introduction

Effective mutagenesis strategies in enzyme engineering are often dependent on the generation of small and targeted, high-quality libraries of mutants[1]. Such ‘smart’ libraries are consistent with practical constraints imposed by the screening effort: while point-mutant libraries are readily screened, we need creative solutions to improve our capacity to explore the combinatorial complexity of sequence space. Indeed, simultaneous amino acid substitutions may have non-additive or epistatic effects (cooperative or antagonistic) on protein function[1–4]. To sample complex mutational patterns, efforts are increasingly made to maximize protein sequence diversity while keeping the library size manageable. Strategies include controlling mutational bias through the use of a reduced genetic alphabet (i.e. NNK, NDT, or more sophisticated methods [5])[6] among other techniques[2, 3, 7, 8]. Advances in computational tools also contribute to establishing semi-rational approaches to enzyme engineering, combining structure-based analysis and computational simulations with elements of randomization[6, 9–14]. Several methods have been described for the generation of smart libraries. These methods typically suffer from one or more disadvantage including high cost, being experimentally demanding, requiring time consuming steps or, importantly, not providing flexibility in recombining mutations. For instance, random mutagenesis is generally restricted to whole-gene randomisation; single-site saturation mutagenesis does not take into account possible synergistic effects of mutations at different sites, and methods that use specialized reagents (i.e. biotinylated primers for ISOR[15]) are expensive. As Kazlauskas and Bornscheuer have pointed out, ‘*the best protein engineering strategy is the one that allows one to reach the goal with the least effort’*[16], hence the need to develop innovative strategies to address a wider range of problems.

This work was inspired by reports of the Golden Gate gene assembly strategy, which has been exploited for the most diverse applications. It is routinely used to assemble genetic parts for synthetic biology applications, such as the recombination of DNA fragments to generate improved plasmid expression systems by linking, in the desired order, components such as promoters, ribosome binding sites, origins of replication, etc. It has also found use in genome engineering to assemble multiple repeat DNA fragments in an orderly fashion to produce TAL effector nucleases[17–20].

Here, we consider applying the method of reassembly within a single gene. While previously reported in the context of shuffling native genes,[19] we apply the strategy to further increase sequence diversity, by combining synthetically mutated gene segments into complex, smart libraries[17, 19, 21–24].

*Candida antarctica lipase A* (Cal-A) was chosen as a model enzyme. Cal-A is an unusual lipase: it is stable at high temperatures (> 90°C) and at acidic pH, accepts sterically hindered and tertiary alcohols as substrates, shows a preference for S_N_2 hydrolysis of triglycerides, offers selectivity towards trans-fatty acids and can accept amino acids and amino esters as substrates[25–27]. Unlike most lipases, its unique interfacial activation mechanism does not involve movement of a big lid domain. Instead, it appears to involve the movement of a short loop which we will refer to as the ‘*small loop’*[26]. This unique combination of features makes Cal-A an ideal target for further development into a valuable industrial lipase with potential for discrimination of short-chain *vs*. long-chain fatty acids, a useful tool for the dairy industry. In fact, studies suggest that milk-fat products rich in diglycerides composed of short-chain saturated fatty acids might have health benefits[28]. The intrinsic selectivity Cal-A for S_N_2 hydrolysis of triglycerides addresses the requirement for diglycerides, while engineering Cal-A to discriminate for the hydrolysis of long *vs*. short-chain fatty acids would, furthermore, allow for selective removal of long-chain fatty acids.

The apo-enzyme structure and the catalytic triad of Cal-A are known[26], yet the mode of binding of its bulky triglyceride substrates remains undefined[25]. To build a ‘smart library’ of Cal-A with sequence diversity throughout the large, putative substrate-binding area, we sought a method allowing seamless assembly of independently mutated gene fragments. The Golden Gate strategy[17, 18] makes use of type IIS restriction enzymes such as *Sap*I and *Bsa*I, which cleave outside of non-palindromic recognition sites such that their recognition site is distinct from their cutting site. *Sap*I recognizes the sequence GCTCTTC and cuts one nucleotide downstream on one strand, and four nucleotides downstream on the opposite strand, leaving a three-base overhang beyond the recognition site. Similarly, *Bsa*I leaves a four-base overhang. Since the overhang is independent from the recognition site, ligation of resulting fragments is ‘scarless’ in that the product no longer contains the recognition site. As a result, the overhangs can be customized to be unique, enabling assembly of multiple fragments simultaneously and in a unidirectional manner[19]. In addition, since the restriction site is absent from the assembled construct, restriction and ligation can be performed in a one-pot fashion.

## Results and discussion

### Library design

Our smart library design considered the reported knowledge on the putative mode of Cal-A substrate binding. The long chain of a C18 fatty acid substrate has been hypothesized to bind in a tunnel where PEG crystalized, and targeted NDT mutagenesis of that region had previously shown some effect on cis-trans substrate selectivity[25]. To explore greater sequence diversity, the entire tunnel region (residues 211–350) was targeted for random mutagenesis and thus constitutes one of the three Cal-A regions we mutated.

Based on further structural analysis (PDB identification code: 2VEO)[26] and proximity to the catalytic triad (within 5 Å from the hydroxyl oxygen of Ser183), residues Tyr93, Tyr183 and Phe431 were selected as potential hot-spots for mutagenesis. Residues Tyr93 and Tyr183 are located in the *N*-terminal region of Cal-A, which we name ‘part 1’. Mutation of the bulky Tyr93, located below the catalytic triad, could modulate substrate access; removal of the bulky Tyr183 appears to open a putative tunnel (confirmed by molecular dynamics simulations; results not shown). Finally, Phe431 is located in ‘part 3’, corresponding to the Cal-A *C*-terminal region. Phe 431 belongs to the ‘small loop’, hypothesized to gate substrate entry [26] (motion observed by molecular dynamics simulations; results not shown). We chose NDT degeneracy at these three positions (where N = A,C,G,T, and D = A, G or T; covering 12 codons, 12 amino acids) as it provides good chemical diversity while keeping library size modest[29].

**Fig 1:**
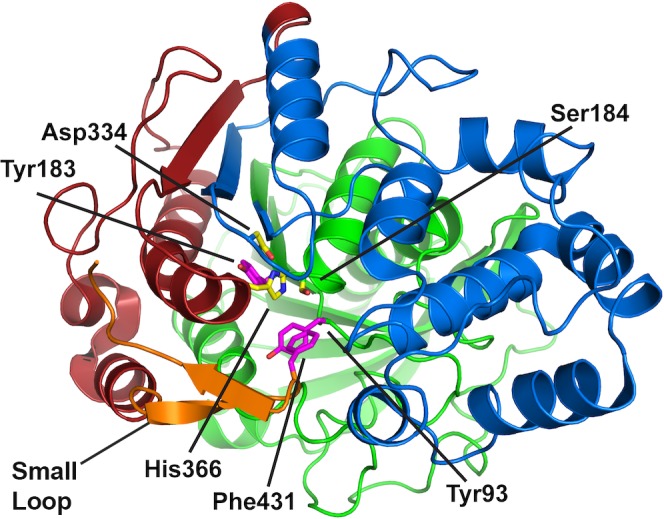
Breakdown of Cal-A into three parts for independent mutagenesis. Shown in cartoon representation with the catalytic triad (Ser184, Asp334, His366; yellow sticks) and key residues (Tyr93, Tyr183 and Phe 431; purple sticks). PART 1 (*N*-terminal region 11–210, in green) is comprised of the α/β fold and includes Tyr93 and Tyr183. PART 2 (tunnel region from 211–350, in blue) has been hypothesized to bind the substrate[25]. PART 3 (from 351 to *C*-terminal His-tag, in red) contains the small loop (in orange), with Phe 431 that may act as a gate-keeping residue. (PDB identification code: 2VEO)[26]

To increase the likelihood of modulating Cal-A substrate specificity, we sought potential synergistic effects that required combining libraries of the above mutations[1, 30]. This strategy integrates random and focused mutagenesis, proposed to be a hallmark of the most successful mutagenic strategies[3]. Our target residues/areas span the entire Cal-A gene (Tyr93, Tyr183, the 211–350 putative tunnel region, and Phe431) and require different mutagenesis methodologies, and are, therefore, ideally suited to being combined for seamless assembly using the Golden Gate method (Fig 1).

The three ‘parts’ of Cal-A were synthesized in a codon-optimized form (S1 Fig) and inserted into the DNA2.0 pM269 mother vector. When ready to be recombined, for example upon library generation, the parts were reassembled into the complete gene in an Electra daughter vector (pD441pelB, DNA2.0) customized to carry the pelB leader sequence for periplasmic expression under control of the T5 promoter (constructs are illustrated in S2 Fig). The pelB signal sequence provides high expression of Cal-A[25]. It is worth noting that it is possible to generate a variety of library combinations: in fact, mutated parts can be recombined with other mutated parts or with the native ones. Each new combination is a library *per se*, and this ensures maximum combinatorial freedom. This method finds an interesting application in the generation of randomized libraries limited to specific parts of a protein. For instance, in our work, it allowed for the generation of a randomized library of part 2 of Cal-A, while maintaining parts 1 and 3 native. This is advantageous when only specific parts of the sequence are deemed worth targeting with random mutagenesis. In this work, it is not known which residues within part 2 of Cal-A are involved in substrate binding, justifying a random approach. The recombination of this randomized library with native parts 1 and 3 ensured minimal potential disruption in the rest of the protein.

**Fig 2:**
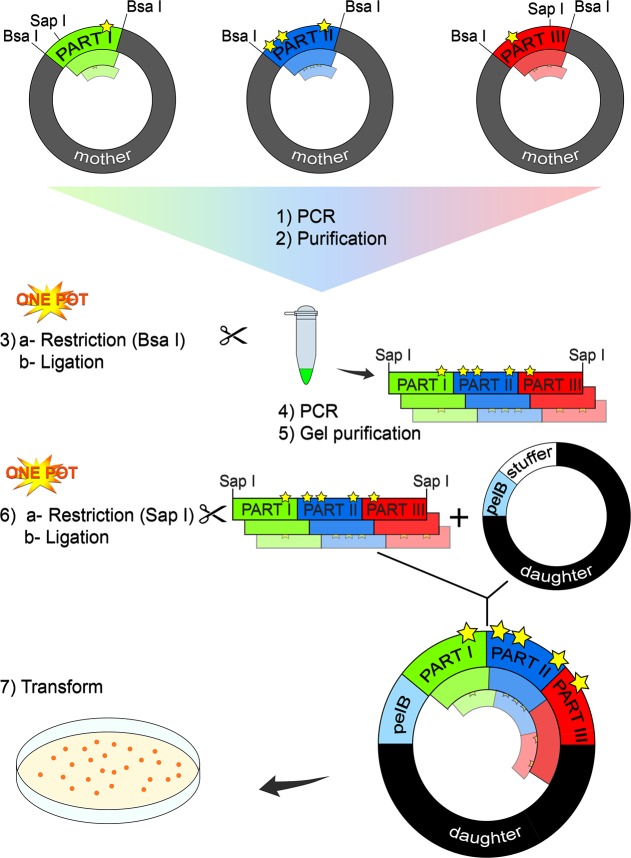
Facile reassembly of individually mutated gene parts. The Cal-A gene was obtained as three separate parts in DNA2.0 mother vectors. In this method, the parts can be mutated independently as appropriate for each part (Table 1, yellow stars represent illustrative mutations). As a proof of concept to demonstrate the versatility of the method, NDT libraries were generated for parts 1 and 3, and part 2 was randomly mutated. The parts (both mutated and wild-type) were then amplified by PCR reactions. They were then purified (steps 1, 2 and S3 Fig) for assembly into a number among the possible combinations of mutated parts (see Table 1 for chosen combinations), in a one-pot restriction-ligation reaction using *Bsa*I (3). The library of assembled genes was PCR amplified and gel purified (4, 5 and S4 Fig). Each amplified library was inserted into the daughter vector (6) using *Sap*I in a one-pot restriction-ligation reaction, for transformation into *E*. *coli* (7). Note that a simplified version of this strategy is also possible, but was found to work only when applied to the wild-type parts (S1 and S5 Figs).

Our assembly strategy used the *Bsa*I and S*ap*I type IIS restriction enzymes, which allowed for the design of customized overhangs. The junctions between parts were designed with a unique *Bsa*I restriction site while two unique *Sap*I restriction sites were designed to ligate the assembled gene into the daughter vector (Fig 2). We note that the parts could equally be ligated into any expression vector containing appropriately designed *Sap*I sites. Furthermore, codon optimization is optional.

The major strength of our method is that the parts can be treated independently for the purposes of library generation, and can be assembled at will to recombine the full gene with any or all parts mutated (primers and conditions are given in S1–S5 Tables). We thus rapidly obtained a variety of complex, mutated libraries, ready to bring forward to screening (Table 1). The final constructs have lost the *Bsa*I and S*ap*I recognition sites. As a consequence, the ligated parts cannot be directly separated by restriction. However, by designing primers with overhangs that reintroduce the *Bsa*I/*Sap*I sites, it is possible to amplify the mutated parts by PCR and mix and match them at any time. Herein rests the impressive flexibility of the system. We note that, as for all cloning strategies, care should be taken to ensure that no unwanted *Bsa*I/*Sap*I restriction sites (or whichever type IIS restriction enzyme is used) are present in the sequence of interest. As discussed above, the diversity included in the ten libraries (Table 1) was generated by mutagenesis using NDT degeneracy for parts 1 and 3 and using error prone PCR for part 2 (S5 Table).

**Table 1:** Mutated Cal-A libraries generated.

	Library name	Library type	Mutagenesis method	Theoretical library size	Variants screened per substrate
**1**	Tyr93	part 1 in mother vector	circular mutagenesis (NDT)	NS	NS
**2**	Tyr183	part 1 in mother vector	circular mutagenesis (NDT)	NS	NS
**3**	Phe431	part 3 in mother vector	circular mutagenesis (NDT)	NS	NS
**4**	Random	part 2 in mother vector	random mutagenesis	NS	NS
**5** ^a^	Tyr93	assembled gene in daughter vector	recombination: part 1* + WT parts 2,3	12	63
**6**	Tyr183	assembled gene in daughter vector	recombination: part 1* + WT parts 2,3	12	48
**7**	Phe431	assembled gene in daughter vector	recombination: part 3* + WT parts 1,2	144	48
**8**	Tyr93/ Phe431	assembled gene in daughter vector	recombination: parts 1*, 3* + WT 2	144	192
**9**	Tyr183/ Phe431	assembled gene in daughter vector	recombination: parts 1*, 3* + WT 2	144	- ^b^
**10**	Random	assembled gene in daughter vector	recombination: part 2* + WT parts 1,3	>10^180^	384

*: mutated parts.

NS: non-screenable constructs.

^a^ Library 5 was generated both in vector pET22b and in mother and daughter vectors. The library size and number of variants screened here refer to the library in pET22b.

^b^ Upon screening library 6, it was recognized that the only substitutions at position 183 that maintain activity are the wild-type Tyr or Phe, hence library 9 was not screened.

The mutants were assembled according to Fig 2. The PCR steps 1 and 4 ensure a high availability of DNA, maximizing the transformation efficiency. Furthermore, when performing restriction and assembling the parts together and with the vector, the total DNA mass in the reaction is reduced if the mother vectors are not present, minimizing reaction volume and units of restriction enzyme needed. Transformation of the mutant libraries yielded at least 10^3^ transformants for each library: between 80 and 100% of the colonies contained the desired constructs. In one instance (construction of library Tyr93-Phe431), use of the circularized daughter plasmid afforded no transformants. This issue was resolved by using the commercial, pre-cut daughter plasmid, although the reason for this difference in performance is not clear.

**Fig 3:**
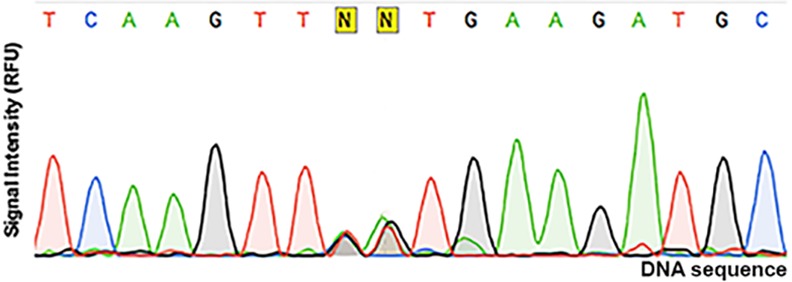
DNA sequencing electropherogram of the Tyr93 mutant library. The peak height is given in relative fluorescence units (RFU) and represents the signal intensity at each nucleotide, along the x axis. The identity of each nucleotide is automatically assigned (above each peak) when the signal is unequivocal, or is labelled ‘N’ when more than one nucleotide provides a statistically significant signal. The NDT degeneracy (25% each A/C/G/T; 33% A/G/T; T) is clearly visible.

Colonies were picked and propagated individually. The quality of each of the degenerate NDT libraries, whether resulting from mutation of one part or assembly of more than one mutated part, was routinely assessed by sequencing pooled clones. As illustrated in Fig 3, codon degeneracy can be clearly visible in the DNA sequencing electropherogram (Fig 3), confirming the expected distribution of nucleotides instead of the original codon. Sequencing of individual clones further confirmed library quality (S6 Table). The quality of the randomly mutated library was assessed by sequencing 20 clones (Table 2). The 20 clones carried a total mutational load of 34, with approximately 25% of the clones being wild-type and the majority of clones carrying two mutations (average = 1.65), for a maximum of 4 mutations per clone.

**Table 2:** Summary of sequencing results defining the quality of the random library (library 10, Table 1).

	**# of mutations per clone**					
	0	1	2	3	4	Total mutations
**# of clones**	5	2	9	2	2	34
	**Properties of mutations**					
	Silent					6
	Stop codon					1
	Transitions/transversions (ratio)					1.1

### Screening

The resulting Cal-A libraries expressed in *E*. *coli* BL21 (DE3) were screened to evaluate selectivity of hydrolase activity toward long versus short-chain triglycerides. To achieve the required screening throughput, we devised a novel strategy based on a well-established *in vivo* screening assay for esterases[31, 32]. Lipases have been screened in the past using robust in-plate assays involving either tributyrin or olive oil/rhodamine emulsions in agar: plates were inoculated with lipase variants, and hydrolytic activity could be detected upon triglyceride hydrolysis either with the formation of a halo of clearance (in the case of tributyrin) or a fluorescent rhodamine halo upon fatty acid release due to a change in pH[31, 33]. Importantly, the aforementioned techniques are well suited to be automated and allow for the screening of the lipase towards triglycerides, which are substrates of industrial interest.

**Fig 4:**
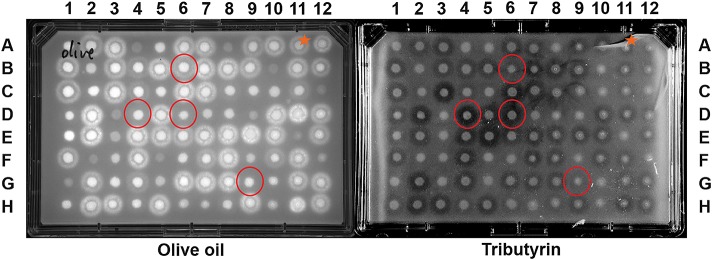
An example set of plates of the randomly-mutated library 10 screened against olive oil/rhodamine (left), and tributyrin (right). Red circles indicate variants able to discriminate between the hydrolysis of short and long chain fatty acids. The fatty acid substrate is emulsified at 2.5% w/v into auto-induction agar medium and rhodamine is at 0.001% w/v. An orange star indicates wild-type Cal-A.

Our strategy makes use of a liquid-handler robot to inoculate individual clones onto rectangular agar plates containing the emulsified triglycerides, starting from saturated cultures of the variants. This results in a perfectly ordered, compact array of variants exposed to the substrate of interest. Either tributyrin (C4), or olive oil (70% oleic acid, C18) with rhodamine, were emulsified into the agar. The inoculated plates were grown at 30°C, overnight (16 hrs) until colonies of manageable size appeared (between 0.2 and 0.5 cm). Active clones gave rise to a clear halo around the colony against the otherwise opaque tributyrin emulsion, or a fluorescent halo in the case of the olive oil and rhodamine emulsion (Fig 4).

The use of a saturated bacterial inoculum (overnight growth) provided highly reproducible results on triplicate plates (S2 Fig). This method allowed us to rapidly and reliably individuate Cal-A lipase variants that discriminate between short- and long-chain triglyceride substrates (Fig 4). It was possible to test four plates of 96 variants at a time. Pictures of the plates were collected using a gel imager. The results were consistent in triplicates (S2 Fig) and analysis of the plates revealed the presence of variants able to discriminate between the various substrates, whilst the wild-type was active with tributyrin and olive oil without distinction. In the representative example of screened plates presented in Fig 4, variant B6 hydrolyzes olive oil preferentially over tributyrin, while variants D4, D6 and G9 show the inverse selectivity. The majority of the variants do not show any improved selectivity compared to the wild-type Cal-A (e.g. A2, A3, the majority of row B, etc, relative to wild-type A11, starred) or they lose the ability to hydrolyze both of the substrates (A1, A4, A5, and so on).

Our protocol allows for screening automation and augments the throughput of an otherwise classical assay for lipase/esterase activity because it arrays whole cells rather than lysates or purified enzyme. The use of a liquid handler ensures reproducibility, precision, and fast operations, making the assay robust and convenient. The rhodamine version of the method is extremely versatile, as oils from the most different sources can be used as substrates for the lipase (i.e. coconut oil, palm oil). A total of 735 clones were screened (Table 1). Among these, 88 clones (12%) showed clear ability to discriminate between long- and short-chain fatty acids whereas the wild-type Cal-A was indiscriminate (Fig 4 and Table 3). When Tyr93 was mutated, 89% of the clones retained activity and discriminated preferentially towards long-chain fatty acids. In contrast, Tyr183 was crucial for activity: when mutated, only 10% of the variants retained activity, with a preference for the hydrolysis of short-chain fatty acids. Phe431 tolerated mutation without little effect, as activity was maintained in 90% of the variants and no increased discrimination was found. For the randomized Part 2 library, 66% of the variants retained activity: the ratio between variants active on short-chain *vs*. long-chain fatty acids was 39:1. These results demonstrate the suitability of the Golden Gate strategy to achieve rapid generation of readily recombined functional diversity.

**Table 3:** Qualitative results of the screening of Cal-A libraries.

Library	Library 5 (Tyr93)	Library 6 (Tyr183)	Library 7 (Phe431)	Library 8 (Tyr93/Phe431)	Library 10 (Random)
**Total variants screened**	63	48	48	192	384
**Active variants** ^a^	56	5	42	84	254
**Inactive variants**	7	43	6	108	130
**Discriminate for short-chain**	0	2	0	17	39
**Discriminate for long-chain**	26	0	0	3	1

^a^ Active variants were defined as such only when halo formation was unequivocal; they were otherwise defined as inactive.

## Conclusions

Our method allows for maximum versatility both in the generation and the screening of smart libraries of mutants. The generation of libraries based on the Golden Gate strategy is quick and enables easy library combination to study the synergistic effect of the mutations. Furthermore, each part of the gene is treated separately, with the possibility of applying different strategies for mutagenesis to each part. Here, we illustrated the use of targeted mutagenesis for parts 1 and 3 and random mutagenesis for part 2. As a result, this method provides a means to create highly mutated portions of genes that can be inserted either into the wild-type background (see Library 10) or recombined together (Libraries 8, 9) such that epistasis among residues within specified regions can readily be explored. Moreover, there is no need to engineer restriction sites into the gene sequence, as the method is seamless. This novel strategy can be applied to any protein, offering countless possibilities for facile generation of smart libraries. Furthermore, the Golden Gate method was previously used to assemble up to nine parts of DNA[19], hence we envisage that the method in this paper could be extended to recombine more than three parts of an enzyme, to achieve even higher flexibility. As a proof of concept, the method was successfully applied in conjunction with a novel automated strategy for qualitative screening to alter the selectivity of Cal-A in hydrolyzing short-chain and long-chain fatty acid esters.

## Materials and methods

### 1. Materials, strains, vectors and culture conditions

Unless otherwise stated, all chemical reagents and DNA primers were purchased as analytical grade from Sigma-Aldrich. Nunc™ OmniTray™ rectangular petri dishes were purchased from Thermo Fisher Scientific. Our *in vivo* screening was performed using a Beckman Coulter Biomek NX^p^ Robot. Restriction enzymes were purchased from New England Biolabs. TAKARA ligase was purchased by Clontech. Protein markers were purchased either from New England Biolabs or Thermofisher. Phusion Green High-Fidelity DNA Polymerase and the PureLink® PCR Purification Kit (Invitrogen) were purchased from Thermo Fisher Scientific. Taq polymerase was purchased from Biobasics. The QuikChange Lightning Site-Directed Mutagenesis Kit was purchased from Agilent Technologies. The GenElute™ Plasmid Miniprep Kit was purchased from Sigma Aldrich. For the generation of the randomly mutated library, the GeneMorph II Random Mutagenesis Kit from Agilent Technologies was used.

Chemically competent *E*. *coli* BL21 (DE3) were prepared by the CaCl_2_ method. The original pelB-Cal-A construct was a kind donation of Prof. Uwe Bornscheuer, Department of Dept. of Biotechnology & Enzyme Catalysis, Greifswald University, Germany. This construct was used to produce the initial Tyr93 NDT library. The library was re-generated in the pM269 DNA2.0 mother vector, and subsequenctly in the pD441pelB daughter vector, as reported below.

The codon-optimized Cal-A parts provided in mother vectors (pM269; chloramphenicol resistant) and the linearized daughter vectors (pD441pelB, pD441OmpA; kanamycin resistant) were purchased from DNA2.0 (California, USA; https://www.dna20.com). The DNA2.0 (https://www.dna20.com) terminology was used throughout this report, where ‘mother vector’ refers to a plasmid carrying a part, and ‘daughter vector’ is the expression plasmid. The codon-optimized sequence of wild-type Cal-A resulting from assembly of the three parts is reported in the supplemental information (S1 Fig). DNA sequencing was performed by the Genomic Platform of IRIC (Institute for Research in Immunology and Cancerology), Université de Montréal, except for sequencing of the Tyr183 and Phe431 libraries that was performed by the Centre d'Innovation Génome Québec at McGill University (QC, Canada).

Transformed *E*. *coli* strains were generally cultured on Luria-Bertani (LB) agar and in LB broth, both containing ampicillin (100 μg/mL), kanamycin (50 μg/mL) or chloramphenicol (35 μg/mL), depending on the resistance marker, at 37°C for 16 hours with shaking at 250 rpm, when appropriate.

### 2. Assembly of plasmids and library generation

Restriction digestion of both daughter plasmids and parts was performed routinely at 37°C over 30 to 45 minutes using either SapI or BsaI restriction enzymes. Ligation of the parts among themselves and with the vector was performed at 37°C, over 30 to 45 minutes. PCR conditions for the amplification of the parts (wild-type and mutated) are detailed in the supplementary information (S2 Table). Mutants of Cal-A were generated by circular mutagenesis according to the manufacturer’s instructions using the QuikChange Lightning Site-Directed mutagenesis kit and mutagenic primers (S1 Table). All genetic constructs were designed and assembled *in silico* using the SnapGene software (GSL Biotech; available at snapgene.com). The mutational frequency and Ts/Tv (transition/transversion ratio) were calculated with the Mutanalyst software, available on line. Library properties calculated at the PEDEL-AA server page[34] assumed a Poisson distribution[35]. The resulting ligation mixes were transformed into *E*.*coli* BL21 (DE3) expression host. Colonies were picked individually and grown in 96 deep-well plates in LB supplemented with the appropriate antibiotic.

### 3. Screening of Cal-A variants through automation by liquid handler robot

The screening of the variants against tributyrin and olive oil was based on previously reported techniques[31, 32]. With the help of a Beckman Coulter Biomek NX^p^ robot, we transformed the manual screen into an automated version that allows for higher throughput. We used (give the dimensions here) rectangular petri dishes to cast the growth media to be inoculated with the library variants. Tributyrin plates were prepared as follows: 1.5 g of agar were added to 100 mL of auto-inducing ZY medium[36] adjusted to pH 8. Tributyrin oil (2.5 g) was added after autoclaving, with kanamycin. For the olive/oil rhodamine plates, the tributyrin was replaced with 2.5 g olive oil, and rhodamine was added at a concentration of 0.001% w/v. The media were thoroughly shaken to generate strong emulsions before plating. Each plate was cast with 42 mL of medium and left to set on a smooth and perfectly horizontal surface. The plates were stacked in the liquid handler, ready to be picked up by the robotic arm. On the deck of the robot, four 96-well plates containing 1 mL aliquots of the library variants pre-grown to saturation (LB, overnight, shaking, 37°C) served as inoculum for the agar plates. A script was designed to pick up 20 μL of culture, column by column, from the inoculum plates and spot 8 μL of inoculum by lightly touching the agar surface. The pipette tips were re-used for the same inoculum. The excess liquid culture was released in an ethanol waste. Inoculated plates were moved by the robotic arm to a second stacker to be picked up by the user for overnight incubation at 30°C. The incubated plates were visualized by a gel imager and analyzed. As a negative control, a culture of *E*. *coli* BL21 (DE3) harboring an inducible, unrelated gene (cytochrome P450 BM3) was tested under the same conditions. A halo of clearance around a colony on tributyrin-containing medium indicates activity toward the short-chain substrate while a halo of fluorescence around a colony on olive oil/rhodamine-containing medium indicates activity toward long-chain substrate. Halo analysis is qualitative, giving a yes/no response as to activity of a variant toward a specific substrate. The method is qualitatively robust, in that an active variant consistently shows a halo and a negative variant consistently does not (S6 Fig = triplicate plates). Although reproducible, the current method does not lend itself to quantitative activity measurements because of factors including insufficient precision in the number of cells inoculated which affects colony size and thus appearance of the halo (diameter and/or intensity).

## Supporting information

S1 Table Primers used in this work. (DOCX)

S2 Table PCR conditions routinely used to amplify the parts from the mother vectors. The Phusion Green High-Fidelity DNA Polymerase was used to ensure maximum fidelity. Primers Inner34_fwd and part1_rvs were used for amplification of part1, part2_fwd and part2_rvs for part2 and part3_fwd and Inner34_rvs for part 3 (S1 Table).
(DOCX)

S3 Table PCR conditions routinely used to amplify the ligated parts before ligation into the daughter vector. Phusion Green High-Fidelity DNA Polymerase was used to ensure maximum fidelity. Primers Inner34_fwd and Inner34_rvs were used for amplification (S1 Table).
(DOCX)

S4 Table Conditions routinely used to perform colony PCR in order to screen for clones carrying the correctly assembled constructs. (DOCX)

S5 Table Conditions used to perform error prone PCR. The random library in part2 was generated using the GeneMorph II Random Mutagenesis Kit by Agilent. This kit allows for the elimination of any bias during mutation. Following the manufacturer’s instructions, we applied the conditions yielding the highest mutation rate. This is achieved by using very little template DNA (0.1 ng) and by repeating the PCR for 30 cycles using primers part2_fwd and part2_rvs (S1 Table). For error-prone PCR to be effective, the DNA yield at the end of the reaction must be between 500 ng and 10 μg. Our reaction yielded 5 μg of DNA, which is within parameters. Once part2 was randomly mutated, we assembled it with the wild-type part1 and part3 and screened for positive clones. Upon screening more than 15 randomly-selected clones for each recombined library, we observed 100% correct ligation products by colony PCR. DNA sequencing of a number of those clones served to assess the library quality (refer to text in main paper and Table 2).
(DOCX)

S6 Table Variants identified upon sequencing libraries with NTD codons. By sequencing approximately 50 clones from each library, we identified 11 of the 12 codons expected for the Tyr93 libraries, 10 of 12 for the Tyr183 libraries and 9 of 12 for the Phe431 libraries.
(DOCX)

S1 Fig DNA2.0 Codon-optimized sequence of wild-type Cal-A upon assembly. (DOCX)

S2 Fig Assembled Cal-A in pD441 daughter vector. The left-hand map shows the entire gene, the right-hand map shows the gene divided into the three parts.
(DOCX)

S3 Fig Representative gel of the three parts extruded from mother vectors. Lane 1: MW, lane 2: PCR product of library 2 (part1), lane 4: PCR product of part 2, lane 6: PCR product of part 3.
(DOCX)

S4 Fig Representative gel of the PCR reaction of the ligated parts. Lanes 1 and 2: PCR product of ligated parts, Lane 4: MW
(DOCX)

S5 Fig One-pot assembly of the wild-type CAL-A. Lane 1: standards, lane 2: plasmid before amplification, lanes 3 to 7: colony PCR product of five randomly chosen clones using primers pFWD and pRVS. The expected band is consistent with the size of the complete Cal-A gene, which is about 1500 bp.
(DOCX)

S6 Fig Example of triplicate results for 96-clone plate screening. Photos of the screening results of 96 variants of the random library against olive oil/rhodamine. The experiment was repeated in triplicate (three plates shown above): the reproducibility of a yes/no response to whether a variant is active or inactive towards olive oil is clear.
(DOCX)

S7 Fig Simplified one-pot assembly procedure. In this simplified one-pot assembly procedure the three mother vectors carrying the parts are restricted and ligated together with the daughter vector in a single reaction (1) and the ligated product is then directly transformed (2). It is worth noting that the use of type IIS restriction enzymes should, in principle, allow to directly cut and ligate the parts and the vector together without the need of PCR steps for pre-amplification. Furthermore, the use of different selective antibiotic markers on the mother and daughter vectors eliminates the worry of carrying forward the mother constructs. In this scenario, the three parts carried in the circular mother vectors are directly pooled with the daughter vector, cut at the respective restriction sites at the same time and assembled by adding the ligase directly to the mixture. We used this simplified assembly strategy during our experiments to generate the wild-type Cal-A construct. Screening by colony PCR (S2 and S4 Tables) showed that the five tested clones contained the desired fragment (S5 Fig). DNA sequencing of three of the clones confirmed that no undesired events had occurred. However, we also noticed that when we applied this simplified strategy to achieve the recombination of the mutant parts, a reduced success rate of ligation was achieved. Even though we cannot at present explain this phenomenon, the use of our standard method (Fig 2 in main article) solved the problem.
(DOCX)
