# Supplementary material for: Enzyme engineering: A synthetic biology approach for more effective library generation and automated high-throughput screening
Source: PLoS One. 2017 Feb 8;12(2):e0171741. doi: 10.1371/journal.pone.0171741 (PMC5298319; doi:10.1371/journal.pone.0171741)
Supplement: S1 Table — (DOCX) [file pone.0171741.s001.docx]

**S1 Table. Primers used in this work.**
