# Supplementary material for: Enzyme engineering: A synthetic biology approach for more effective library generation and automated high-throughput screening
Source: PLoS One. 2017 Feb 8;12(2):e0171741. doi: 10.1371/journal.pone.0171741 (PMC5298319; doi:10.1371/journal.pone.0171741)
Supplement: S3 Table — Phusion Green High-Fidelity DNA Polymerase was used to ensure maximum fidelity. Primers Inner34_fwd and Inner34_rvs were used for amplification (S1 Table). (DOCX) [file pone.0171741.s003.docx]

**S3 Table. PCR conditions routinely used to amplify the ligated parts before ligation into the daughter vector**.

| Reagents | amount |  |
| --- | --- | --- |
| 5x Phusion Green buffer | 10 µL |  |
| 10 mM dNTPmix | 1 µL |  |
| Inner34_fwd primer 10 µM | 0.5 µL |  |
| Inner34_rvs primer 10 µM | 0.5 µL |  |
| template | 10 µL of ligation product |  |
| milliQ water | to 50 µL |  |
| Phusion polymerase | 0.5 µL |  |
|  |  |  |
| Cycles | time | temperature |
| 1 cycle | 2 min 30 sec | 98 °C |
| 35 cycles | 10 sec | 98 °C |
|  | 1 min 10 sec | 72 °C |
|  | 10 min | 72 °C |
| hold | ∞ | 4°C |

Phusion Green High-Fidelity DNA Polymerase was used to ensure maximum fidelity. Primers Inner34_fwd and Inner34_rvs were used for amplification (S1 Table).
