# Supplementary material for: Enzyme engineering: A synthetic biology approach for more effective library generation and automated high-throughput screening
Source: PLoS One. 2017 Feb 8;12(2):e0171741. doi: 10.1371/journal.pone.0171741 (PMC5298319; doi:10.1371/journal.pone.0171741)
Supplement: S4 Table — (DOCX) [file pone.0171741.s004.docx]

**S4 Table. Conditions routinely used to perform colony PCR in order to screen for clones carrying the correctly assembled constructs.**

| Reagent: | amount |  |
| --- | --- | --- |
| 10 x Taq buffer | 5 µL |  |
| 10 mM dNTPmix | 1 µL |  |
| pFWD primer 10 µM | 2 µL |  |
| pRVS primer 10 µM | 2 µL |  |
| template | 1 colony |  |
| milliQ water | to 50 µL |  |
| Taq polymerase | 0.25 µL |  |
|  |  |  |
| Cycles | time | temperature |
| 1 cycle | 5 min | 98 °C |
| 25 cycles | 20 sec | 95 °C |
|  | 40 sec | 59 °C |
|  | 1 min 30 sec | 72 °C |
| 1 cycle | 5 min | 72 °C |
| hold | ∞ | 4°C |
