# Supplementary material for: Enzyme engineering: A synthetic biology approach for more effective library generation and automated high-throughput screening
Source: PLoS One. 2017 Feb 8;12(2):e0171741. doi: 10.1371/journal.pone.0171741 (PMC5298319; doi:10.1371/journal.pone.0171741)
Supplement: S5 Table — The random library in part2 was generated using the GeneMorph II Random Mutagenesis Kit by Agilent. This kit allows for the elimination of any bias during mutation. Following the manufacturer’s instructions, we applied the conditions yielding the highest mutation rate. This is achieved by using very little template DNA (0.1 ng) and by repeating the PCR for 30 cycles using primers part2_fwd and part2_rvs (S1 Table). For error-prone PCR to be effective, the DNA yield at the end of the reaction must be between 500 ng and 10 μg. Our reaction yielded 5 μg of DNA, which is within parameters. Once part2 was randomly mutated, we assembled it with the wild-type part1 and part3 and screened for positive clones. Upon screening more than 15 randomly-selected clones for each recombined library, we observed 100% correct ligation products by colony PCR. DNA sequencing of a number of those clones served to assess the library quality (refer to text in main paper and Table 2). (DOCX) [file pone.0171741.s005.docx]

**S5 Table. Conditions used to perform error prone PCR.**

| Reagent: | amount |  |
| --- | --- | --- |
| 10x Mutazyme II reaction buffer | 10 µL |  |
| primers mix (250 ng/µL each) | 0.5 µL |  |
| template | 0.1 ng |  |
| milliQ water | to 50 µL |  |
| Mutazyme II DNA polymerase | 1 µL |  |
|  |  |  |
| Cycles | time | temperature |
| 1 cycle | 2 min | 95 °C |
| 30 cycles | 30 sec | 95 °C |
|  | 30 sec | 55 °C |
|  | 1 min | 72 °C |
|  | 10 min | 72 °C |
| hold | ∞ | 4°C |

The random library in part2 was generated using the GeneMorph II Random Mutagenesis Kit by Agilent. This kit allows for the elimination of any bias during mutation. Following the manufacturer’s instructions, we applied the conditions yielding the highest mutation rate. This is achieved by using very little template DNA (0.1 ng) and by repeating the PCR for 30 cycles using primers part2_fwd and part2_rvs (S1 Table). For error-prone PCR to be effective, the DNA yield at the end of the reaction must be between 500 ng and 10 µg. Our reaction yielded 5 µg of DNA, which is within parameters. Once part2 was randomly mutated, we assembled it with the wild-type part1 and part3 and screened for positive clones. Upon screening more than 15 randomly-selected clones for each recombined library, we observed 100% correct ligation products by colony PCR. DNA sequencing of a number of those clones served to assess the library quality (refer to text in main paper and Table 2).
