# Supplementary material for: Enzyme engineering: A synthetic biology approach for more effective library generation and automated high-throughput screening
Source: PLoS One. 2017 Feb 8;12(2):e0171741. doi: 10.1371/journal.pone.0171741 (PMC5298319; doi:10.1371/journal.pone.0171741)
Supplement: S6 Table — By sequencing approximately 50 clones from each library, we identified 11 of the 12 codons expected for the Tyr93 libraries, 10 of 12 for the Tyr183 libraries and 9 of 12 for the Phe431 libraries. (DOCX) [file pone.0171741.s006.docx]

**S6 Table. Variants identified upon sequencing libraries with NTD codons**

**
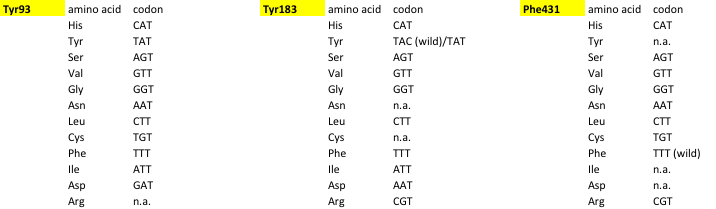
**

By sequencing approximately 50 clones from each library, we identified 11 of the 12 codons expected for the Tyr93 libraries, 10 of 12 for the Tyr183 libraries and 9 of 12 for the Phe431 libraries.
