# Supplementary material for: Enzyme engineering: A synthetic biology approach for more effective library generation and automated high-throughput screening
Source: PLoS One. 2017 Feb 8;12(2):e0171741. doi: 10.1371/journal.pone.0171741 (PMC5298319; doi:10.1371/journal.pone.0171741)
Supplement: S1 Fig — (DOCX) [file pone.0171741.s007.docx]

**S1 Figure. DNA2.0 Codon-optimized sequence of wild-type Cal-A upon assembly**

ATGAAATACCTATTGCCTACGGCAGCCGCTGGATTGTTATTACTCGCTGCCCAACCAGCCATGGCGATGGCCGCTCTGCCTAACCCGTACGATGATCCTTTTTACACCACCCCGTCCAACATTGGCACGTTCGCCAAGGGCCAGGTCATTCAGAGCCGCAAGGTTCCGACGGACATTGGTAACGCAAACAACGCAGCGAGCTTCCAACTGCAGTATCGTACGACCAATACCCAGAATGAAGCTGTCGCCGATGTGGCGACGGTTTGGATTCCAGCCAAACCGGCTTCTCCGCCGAAAATCTTCAGCTATCAAGTTTATGAAGATGCGACCGCGCTGGACTGCGCACCGAGCTATTCCTACCTCACCGGTCTGGACCAGCCGAACAAAGTTACCGCGGTTCTGGACACGCCGATTATCATCGGTTGGGCGCTGCAGCAAGGTTACTATGTCGTTAGCAGCGACCACGAGGGCTTTAAAGCCGCGTTCATCGCGGGCTACGAAGAGGGCATGGCCATCTTGGACGGTATTCGCGCATTGAAGAATTACCAGAATCTGCCTAGCGATAGCAAAGTCGCTCTGGAAGGCTACTCTGGTGGCGCGCATGCAACGGTCTGGGCGACTAGCCTGGCGGAGAGCTATGCGCCGGAACTGAATATTGTGGGTGCGTCCCATGGTGGCACCCCGGTGAGCGCAAAAGATACGTTCACCTTCCTGAATGGTGGCCCATTTGCCGGCTTCGCCCTGGCAGGCGTGAGCGGCCTGTCGCTGGCGCACCCGGACATGGAATCTTTCATCGAAGCGCGTCTGAACGCAAAGGGTCAACGTACGCTGAAGCAAATCCGTGGTCGCGGCTTTTGCTTGCCGCAAGTCGTGCTGACCTACCCGTTTTTGAACGTTTTTAGCCTGGTCAATGATACCAACTTGCTGAATGAAGCACCGATCGCGAGCATTCTGAAACAAGAGACTGTCGTGCAGGCTGAGGCTTCCTACACCGTGTCCGTGCCGAAGTTTCCGCGTTTCATCTGGCACGCAATCCCGGACGAAATCGTACCGTATCAGCCGGCGGCGACGTACGTTAAAGAACAGTGTGCGAAGGGTGCGAACATCAATTTTAGCCCGTATCCGATTGCCGAGCACCTGACGGCAGAGATCTTCGGTCTGGTTCCGAGCTTATGGTTCATTAAACAAGCATTCGATGGTACCACGCCGAAAGTGATCTGTGGTACCCCAATTCCGGCGATTGCGGGTATCACCACCCCGAGCGCGGATCAAGTTCTGGGTAGCGACCTGGCTAACCAGCTGCGTAGCCTGGACGGCAAACAGAGCGCGTTTGGTAAGCCGTTTGGTCCGATTACTCCGCCAGCAGCGGCACTGGAGCACCATCATCACCACCACTAA
