# Supplementary material for: Enzyme engineering: A synthetic biology approach for more effective library generation and automated high-throughput screening
Source: PLoS One. 2017 Feb 8;12(2):e0171741. doi: 10.1371/journal.pone.0171741 (PMC5298319; doi:10.1371/journal.pone.0171741)
Supplement: S2 Fig — The left-hand map shows the entire gene, the right-hand map shows the gene divided into the three parts. (DOCX) [file pone.0171741.s008.docx]

**S2 Figure. Assembled Cal-A in pD441 daughter vector. The left-hand map shows the entire gene, the right-hand map shows the gene divided into the three parts.**

**
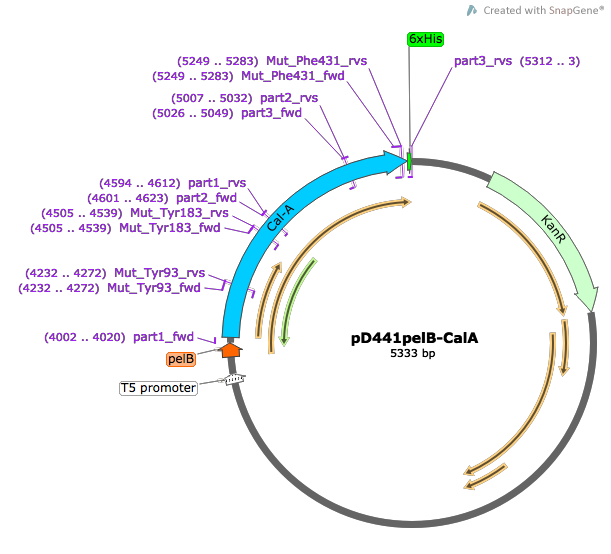

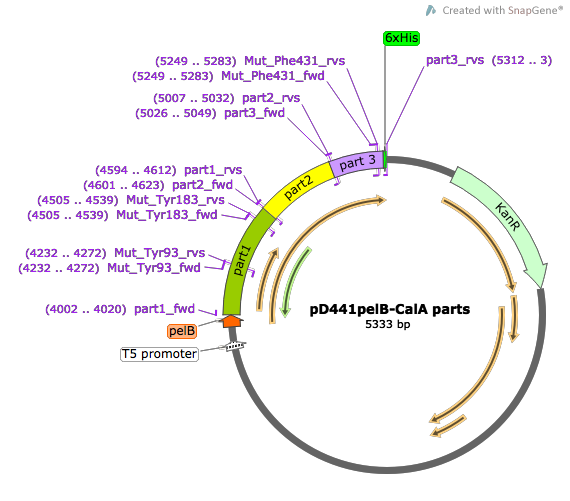
**
