# Supplementary material for: Enzyme engineering: A synthetic biology approach for more effective library generation and automated high-throughput screening
Source: PLoS One. 2017 Feb 8;12(2):e0171741. doi: 10.1371/journal.pone.0171741 (PMC5298319; doi:10.1371/journal.pone.0171741)
Supplement: S3 Fig — Lane 1: MW, lane 2: PCR product of library 2 (part1), lane 4: PCR product of part 2, lane 6: PCR product of part 3. (DOCX) [file pone.0171741.s009.docx]

**S3 Figure. Representative gel of the three parts extruded from mother vectors.**


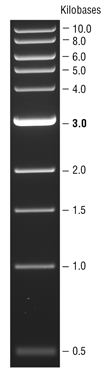

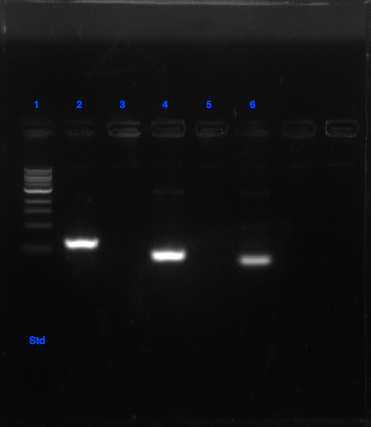


Lane 1: MW, lane 2: PCR product of library 2 (part1), lane 4: PCR product of part 2, lane 6: PCR product of part 3.
