# Supplementary material for: Enzyme engineering: A synthetic biology approach for more effective library generation and automated high-throughput screening
Source: PLoS One. 2017 Feb 8;12(2):e0171741. doi: 10.1371/journal.pone.0171741 (PMC5298319; doi:10.1371/journal.pone.0171741)
Supplement: S4 Fig — Lanes 1 and 2: PCR product of ligated parts, Lane 4: MW (DOCX) [file pone.0171741.s010.docx]

**S4 Figure. Representative gel of the PCR reaction of the ligated parts.**


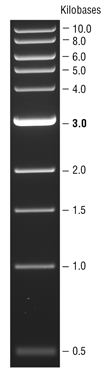
**
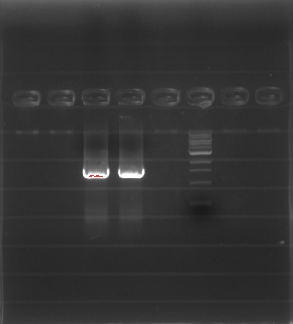
**

Lanes 1 and 2: PCR product of ligated parts, Lane 4: MW
