# Supplementary material for: Enzyme engineering: A synthetic biology approach for more effective library generation and automated high-throughput screening
Source: PLoS One. 2017 Feb 8;12(2):e0171741. doi: 10.1371/journal.pone.0171741 (PMC5298319; doi:10.1371/journal.pone.0171741)
Supplement: S5 Fig — Lane 1: standards, lane 2: plasmid before amplification, lanes 3 to 7: colony PCR product of five randomly chosen clones using primers pFWD and pRVS. The expected band is consistent with the size of the complete Cal-A gene, which is about 1500 bp. (DOCX) [file pone.0171741.s011.docx]

**S5 Figure.** **One-pot assembly of the wild-type CAL-A.**


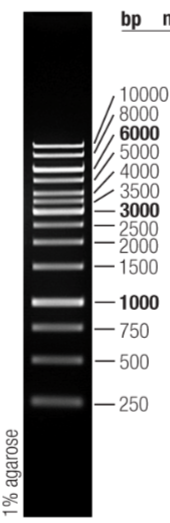

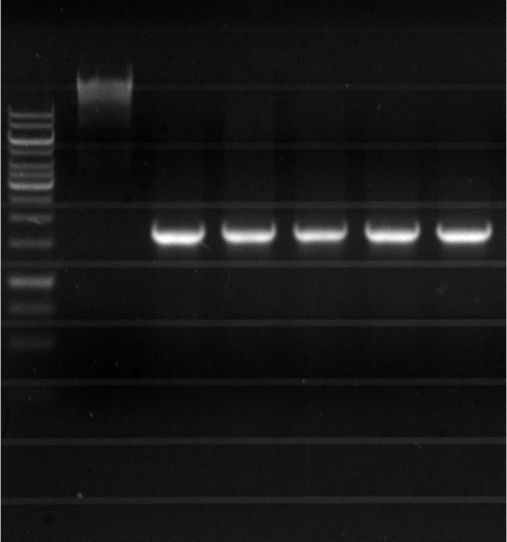


Lane 1: standards, lane 2: plasmid before amplification, lanes 3 to 7: colony PCR product of five randomly chosen clones using primers pFWD and pRVS. The expected band is consistent with the size of the complete Cal-A gene, which is about 1500 bp.
