# Supplementary material for: Enzyme engineering: A synthetic biology approach for more effective library generation and automated high-throughput screening
Source: PLoS One. 2017 Feb 8;12(2):e0171741. doi: 10.1371/journal.pone.0171741 (PMC5298319; doi:10.1371/journal.pone.0171741)
Supplement: S6 Fig — Photos of the screening results of 96 variants of the random library against olive oil/rhodamine. The experiment was repeated in triplicate (three plates shown above): the reproducibility of a yes/no response to whether a variant is active or inactive towards olive oil is clear. (DOCX) [file pone.0171741.s012.docx]

**S6 Fig. Example of triplicate results for 96-clone plate screening.**

**
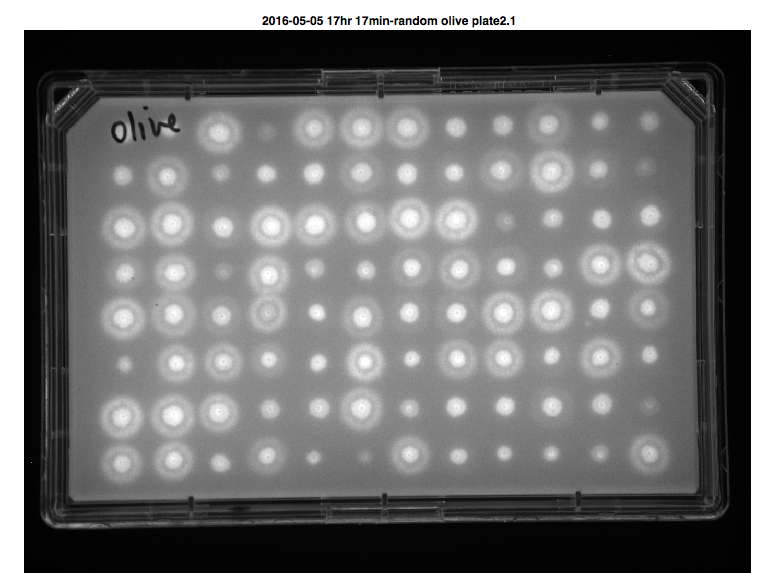
** **
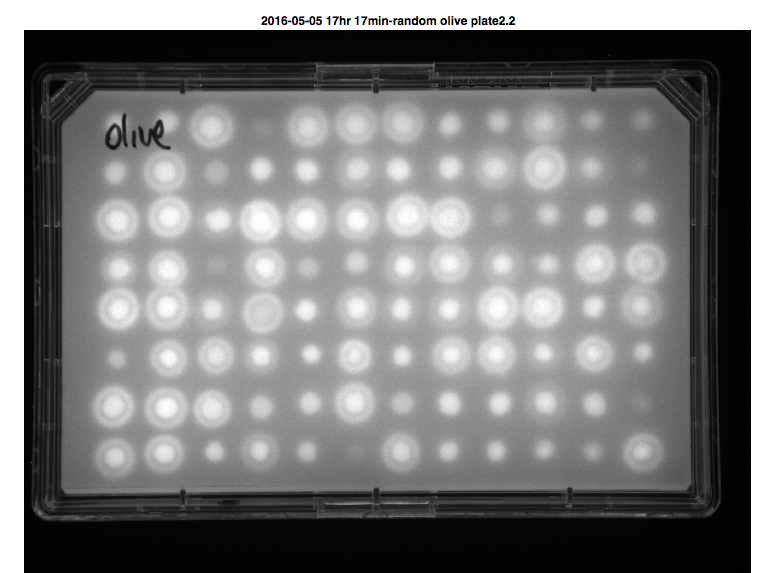
**
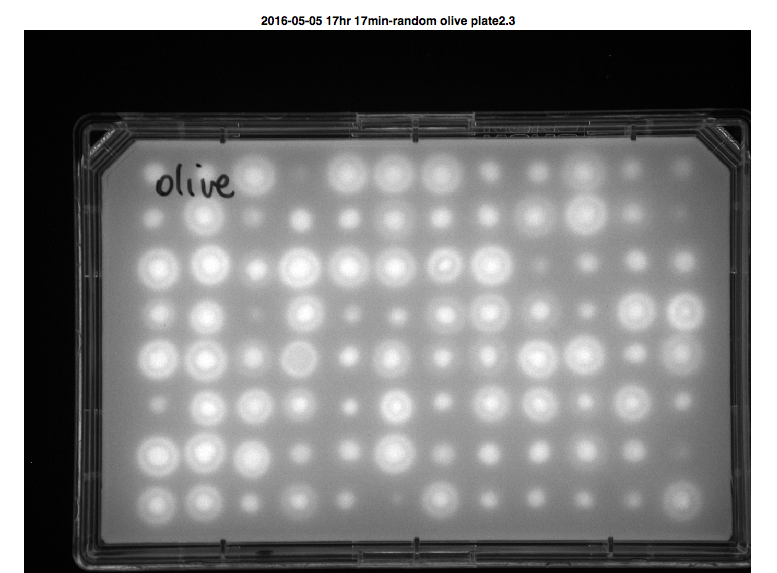


Photos of the screening results of 96 variants of the random library against olive oil/rhodamine. The experiment was repeated in triplicate (three plates shown above): the reproducibility of a yes/no response to whether a variant is active or inactive towards olive oil is clear.
