# Supplementary material for: Enzyme engineering: A synthetic biology approach for more effective library generation and automated high-throughput screening
Source: PLoS One. 2017 Feb 8;12(2):e0171741. doi: 10.1371/journal.pone.0171741 (PMC5298319; doi:10.1371/journal.pone.0171741)
Supplement: S7 Fig — In this simplified one-pot assembly procedure the three mother vectors carrying the parts are restricted and ligated together with the daughter vector in a single reaction (1) and the ligated product is then directly transformed (2). It is worth noting that the use of type IIS restriction enzymes should, in principle, allow to directly cut and ligate the parts and the vector together without the need of PCR steps for pre-amplification. Furthermore, the use of different selective antibiotic markers on the mother and daughter vectors eliminates the worry of carrying forward the mother constructs. In this scenario, the three parts carried in the circular mother vectors are directly pooled with the daughter vector, cut at the respective restriction sites at the same time and assembled by adding the ligase directly to the mixture. We used this simplified assembly strategy during our experiments to generate the wild-type Cal-A construct. Screening by colony PCR (S2 and S4 Tables) showed that the five tested clones contained the desired fragment (S5 Fig). DNA sequencing of three of the clones confirmed that no undesired events had occurred. However, we also noticed that when we applied this simplified strategy to achieve the recombination of the mutant parts, a reduced success rate of ligation was achieved. Even though we cannot at present explain this phenomenon, the use of our standard method (Fig 2 in main article) solved the problem. (DOCX) [file pone.0171741.s013.docx]

**S7 Figure. Simplified one-pot assembly procedure.** In this simplified one-pot assembly procedure the three mother vectors carrying the parts are restricted and ligated together with the daughter vector in a single reaction (1) and the ligated product is then directly transformed (2). It is worth noting that the use of type IIS restriction enzymes should, in principle, allow to directly cut and ligate the parts and the vector together without the need of PCR steps for pre-amplification. Furthermore, the use of different selective antibiotic markers on the mother and daughter vectors eliminates the worry of carrying forward the mother constructs. In this scenario, the three parts carried in the circular mother vectors are directly pooled with the daughter vector, cut at the respective restriction sites at the same time and assembled by adding the ligase directly to the mixture.

We used this simplified assembly strategy during our experiments to generate the wild-type Cal-A construct. Screening by colony PCR (Tables S2 and S4) showed that the five tested clones contained the desired fragment (Figure S5). DNA sequencing of three of the clones confirmed that no undesired events had occurred.

However, we also noticed that when we applied this simplified strategy to achieve the recombination of the mutant parts, a reduced success rate of ligation was achieved. Even though we cannot at present explain this phenomenon, the use of our standard method (Fig 2 in main article) solved the problem.

**
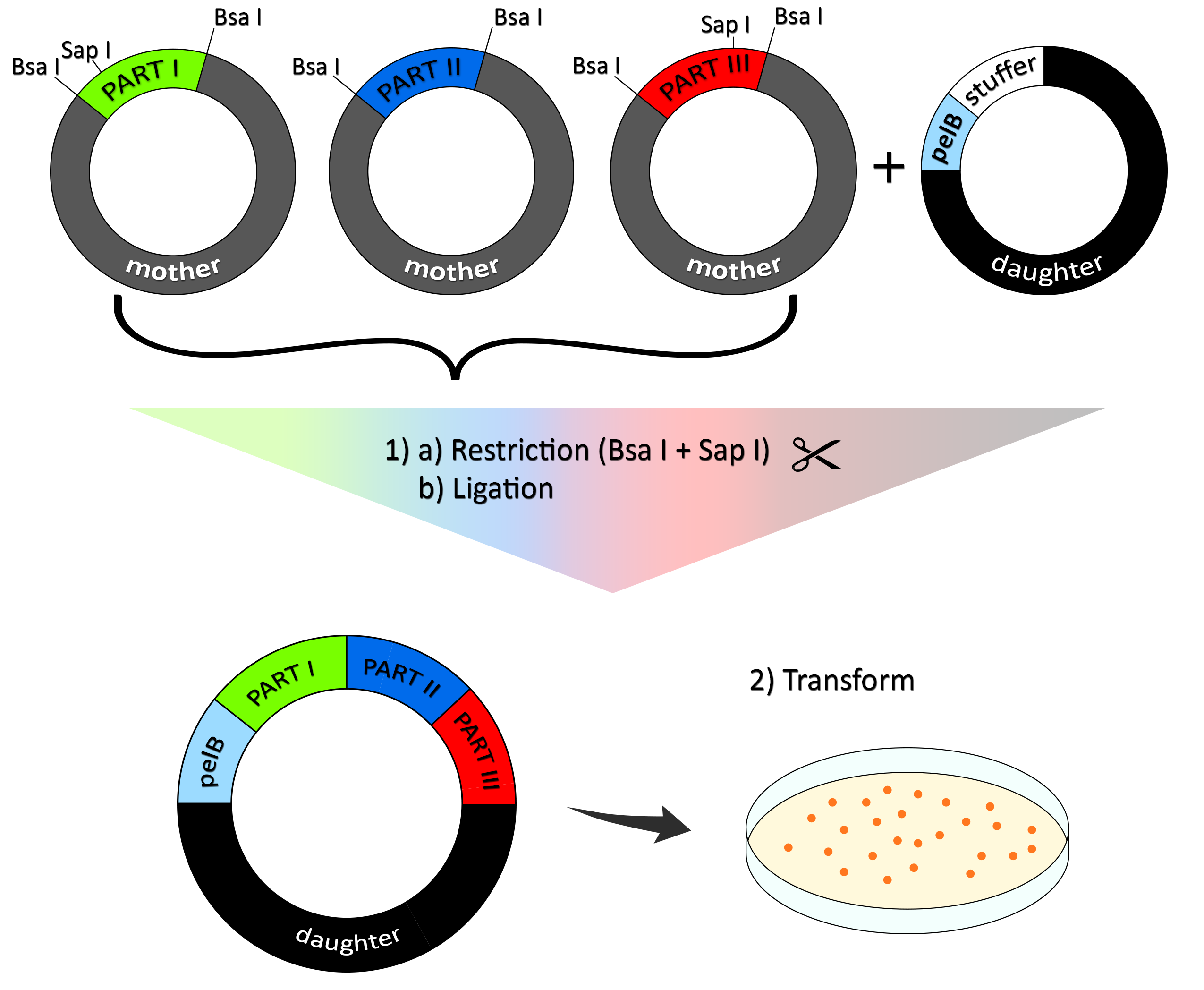
**
